# Sarcomatoid Carcinoma Metastasis to the Colon from a Small Renal Mass: Case Report with Review of Literature

**DOI:** 10.15586/jkcvhl.v10i4.297

**Published:** 2023-10-31

**Authors:** Shailesh Patidar, Arun Ramdas Menon, Shirley Sundersingh, Ramakrishnan Ayloor Seshadri, Anand Raja

**Affiliations:** Department of Surgical Oncology, Cancer Institute (WIA), Chennai, India

**Keywords:** metastasis from small renal mass, renal cell carcinoma, sarcomatoid differentiation, solitary metastasis, synchronous metastasis in RCC

## Abstract

A third of patients with renal cell carcinoma (RCC) present with metastatic disease. Metastasis in RCC from small renal mass (SRM) (≤4 cm) is rare. We report a case of stage cT1a clear-cell RCC with low-risk features on pathology presenting with disproportionately large synchronous solitary metastasis to the transverse colon. He underwent resection of the mass with the involved transverse colon and adjoining mesocolon. Intestinal continuity was restored, following which partial nephrectomy was performed for the right renal tumor. Final pathology of the right renal mass confirmed clear-cell RCC. The large mass after immunohistochemistry profile confirmed metastasis from the renal tumor.

## Introduction

A third of patients with renal cell carcinoma (RCC) present with metastatic disease (1, 2). Although metastasis occurs most frequently to lung, bone, liver, and lymph nodes, RCC is notorious for metastasis to unusual sites. Metastases in RCC characterized as small renal mass (SRM) (≤4 cm) is rare, with reports of synchronous and metachronous disease in 0.9–2.3% and 2.2–2.4%, respectively (3–5). Increased risk of metastasis has been reported in stage T1a tumors with size ≥3 cm, adverse pathology such as Fuhrman’s nuclear grade 3 or 4, or sarcomatoid differentiation (3). We report a case of stage T1a clear-cell RCC with low-risk features on pathology presenting with a disproportionately large synchronous solitary metastasis to the transverse colon.

## Case Report

**Figure 1:**
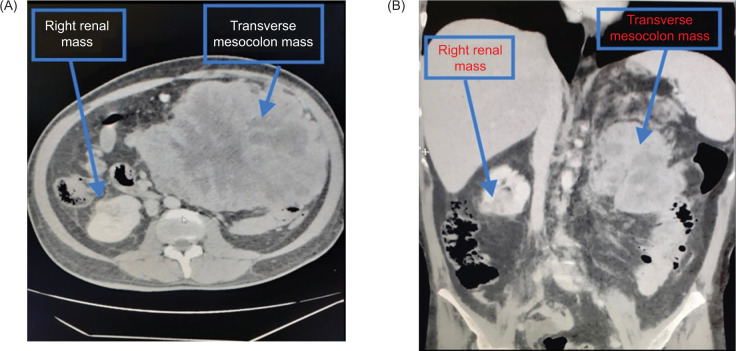
(A–B) Contrast-enhanced CT showing heterogeneously enhancing mass adjacent to the transverse colon and right lower pole kidney mass.

A 40-year-old male presented to us with worsening abdominal pain of 1 month duration associated with intermittent low-grade fever. Examination revealed a non-tender, large mobile epigastric mass. Routine hematology and biochemistry were normal except for erythrocyite sedimentation rate and C-reactive protein (CRP), which were elevated, 100 mm hour^−1^ and 173.8 mg L^−1^, respectively. Blood and urine cultures were negative. Contrast-enhanced computed tomography (CT) of the abdomen showed a 20 cm heterogeneously enhancing mass adjacent to the transverse colon (Figure 1A, 1B). A 2.5 cm enhancing mass was also noted in the lower pole of the right kidney (Figure 1A, 1B).

Transcutaneous biopsy of the mass showed a sarcomatoid tumor, and biopsy of the SRM showed clear-cell renal cell carcinoma. Positron emission tomography/CT did not reveal additional sites of disease.

**Figure 2:**
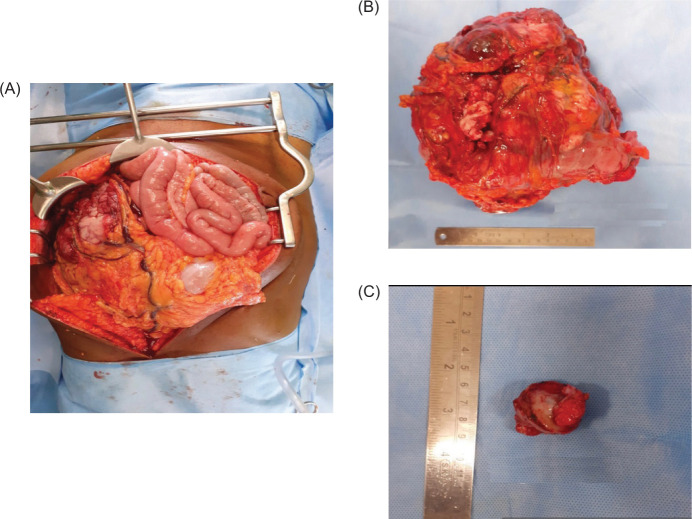
(A) Intraoperative image showing large mass adjacent to transverse colon. (B) Resected specimen of transverse colon mass. (C) Resected specimen of right renal mass.

We proceeded with surgery. Intraoperatively, the mass was noted to infiltrate the distal transverse colon and splenic flexure and extend into the mesocolon. The mass abutted the duodeno-jejunal flexure of small intestine but did not infiltrate it. Resection of the mass with the involved transverse colon and adjoining mesocolon was performed. Intestinal continuity was restored, following which partial nephrectomy was performed for the right renal tumor. Figure 2A-C shows intraoperative image of the mass and resected specimen.

**Figure 3:**
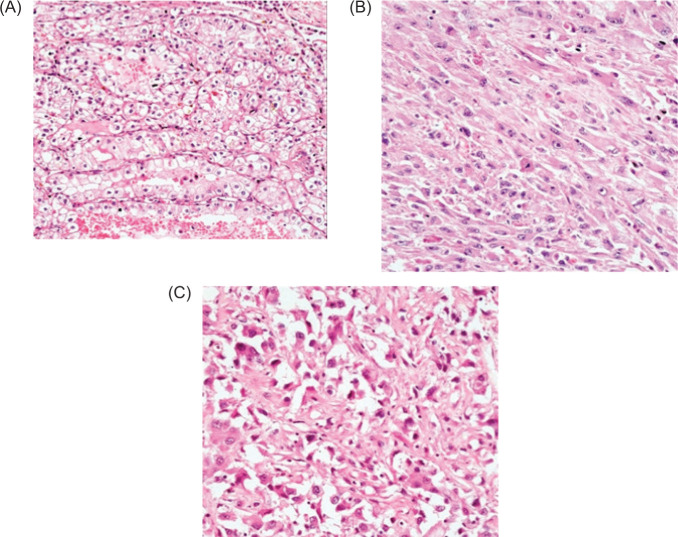
(A) Renal tumor showing glands lined by tumor cells with clear cytoplasm, well defined cell borders, and vesicular nuclei with small nucleoli. H&E 200×. (B) Spindle-shaped tumor cells show abundant cytoplasm with vesicular to hyperchromatic nuclei with prominent nucleoli and increased mitosis. H&E 200×. (C) Tumor cells with rhabdoid morphology shows hyperchromatic, pleomorphic nuclei with prominent nucleoli. H&E 200×.

Final pathology of the right renal mass confirmed clear-cell RCC (Figure 3A). The large mass showed neoplasm composed of cells arranged in sheets, with abundant cytoplasm with vesicular to hyperchromatic nuclei with prominent nucleoli (Figure 3B). Sarcomatoid and rhabdoid features were noted with areas of necrosis (Figure 3C).

**Figure 4:**
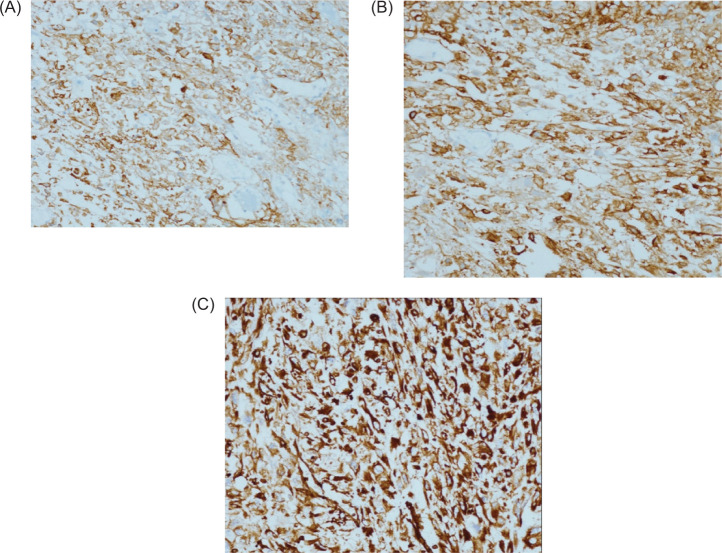
(A) Tumor cells express CD 10. DAB 200×. (B) Tumor cells express Carbonic Anhydrase 9. DAB 200×. (C) Tumor cells express vimentin. DAB 200×.

The tumor was seen to involve the serosa and muscularis propria of the colon, with sparing of the mucosa. Immunohistochemistry profile, positive for CD 10 (Figure 4A), Carbonic Anhydrase 9 (Figure 4B), Vimentin (Figure 4C), and Keratin, confirmed metastasis from the renal tumor.

After discussion in a multidisciplinary meeting, he was advised immune check point inhibitor therapy, which is the standard of care in RCC with sarcomatoid differentiation. However, due to financial constraints, he was initiated on pazopanib. At the time of submission of this case report, the patient remains disease free at 22 months of follow-up.

## Discussion

With increased use of cross-sectional imaging, there has been an exponential increase in the diagnosis of SRM over the last two decades (6). A substantial proportion of these tumors are of low metastatic potential, with questionable capacity to alter the patient’s overall survival (6). Hence, recent attention has been directed at the prevention of overtreatment of these tumors (7). However, there exists a subset of SRM that is of aggressive biology with ability to metastasize, as in our patient. Our report is noteworthy for several reasons – the small size of the primary tumor, the unusual site of metastasis, and the presence of sarcomatoid differentiation in the metastasis despite its absence in the primary renal tumor.

Our patient’s tumor on biopsy and extirpative pathology showed clear-cell RCC with Fuhrman II nuclear grade, which would qualify as a “low-risk” RCC (6). This emphasizes that all RCC, even small tumors without aggressive histology, can metastasize. The risk of metastasis from RCC ≤ 4 cm has been the subject of study of a hand full of articles. A recent SEER analysis showed overall synchronous metastasis in T1 RCC of 2.3% (3), with rates dependent on tumor size, histologic subtype, and grade. The highest rates were noted in the collecting duct and sarcomatoid tumors (7.6–49.1%), followed by clear-cell RCC with nuclear grade 3–4 with rates ranging from 1.2 to 8.9% (3). In addition to these, Takayama et al. reported symptomatic presentation, CRP > 0.4 mg/dL, and microvascular invasion as factors associated with metastasis in SRM (4). The authors also reported a cut off tumor size of ≥3.0 cm as a significant risk factor for metastasis. Similarly, Lee et al. reported a positive linear relationship between increasing tumor size and the likelihood of metastasis, with no patient with tumor size ≤1.0 cm in their series developing metastasis (5).

Other factors reported to be associated with metastasis in SRM are low performance status, symptomatic presentation, male gender, increasing age, pT3 stage and worse renal function (8–10).

Our patient’s primary renal tumor had none of the preceding aggressive pathologic features. Based on the biopsy pathology, he would have been a candidate for active surveillance, if not for the presence of metastatic disease.

Although gastrointestinal metastasis from RCC do occur, albeit infrequently, these are usually associated with concomitant widespread metastatic disease at other sites as well (11, 12). Solitary isolated metastasis to the gastrointestinal tract is rare. In an autopsy series of 687 histology proven RCC cases, metastasis to the gastrointestinal tract was noted in 9.6%, with colon or mesentery involvement noted in only 4 (0.5%) and 11 (1.6%) patients (12). Patients with intestinal metastasis can present with gastrointestinal bleeding, intussusception, bowel obstruction, or biliary obstruction (11). Despite the large size of metastasis, our patient did not have gastrointestinal symptoms, likely due lack of mucosal involvement or luminal compromise. To our knowledge, ours is the first report of SRM metastasis to colon.

Sarcomatoid differentiation likely represents the end stage of RCC dedifferentiation and is typically noted in large tumors, with occurrence ranging from 1 to 32% depending on RCC subtype (13, 14). It occurs in T1a RCC in approximately 0.5% cases, and as with larger tumors, is strongly associated with synchronous and metachronous metastasis (3, 4). Pecoraro reported a metastatic rate of 36.2% has been reported for T1 RCC with sarcomatoid features (3). Similarly, Takayama et al. reported a 15-fold increase in the hazard risk for recurrence for tumors with a sarcomatoid component compared to those without (4). In our patient, the presence of sarcomatoid differentiation in the metastasis, without its presence in the primary kidney tumor, is remarkable. We hypothesize that the metastatic lesion may have developed denovo sarcomatoid dedifferentiation, likely due to its large size, as no sarcomatoid component or aggressive histologic features were identified in the primary renal tumor despite a thorough search by our pathologist.

The management of RCC with sarcomatoid differentiation is an area of active research, with proponents for targeted therapy, immune check point inhibitors, chemotherapy, or combinations of these (13). In metastatic sarcomatoid RCC, immune checkpoint inhibitor (ICI) is a valid option and results in better outcomes compared to traditional therapy with molecular-targeted agents or chemotherapy. These results are consistent with those reported by Raychaudhuri et al. in their case series of two patients with sarcomatoid RCC, who had prolonged responses to nivolumab following failure of tyrosine kinase inhibitor (TKI) therapy (15). The cellular expression of PD-L1 in sarcomatoid RCC was characterized by Joseph et al. in 40 patients and found PD-L1 positivity in 89% of samples (16). In addition, the sarcomatoid component in RCC is known to have a greater mutation burden in terms of higher number of somatic single nucleotide variants and increased frequency of loss of heterozygosity, compared to the carcinomatous component (17). These findings suggest the immunosuppressive nature of sarcomatoid RCC and its potential susceptibility to ICI. TKI monotherapy is currently administered as an alternative strategy as first-line treatment if the patient cannot tolerate or undergo immunotherapy. In TKI era, sunitinib was among the first TKIs approved to treat metastatic RCC (18). Although ICIs are considered standard of care in metastatic RCC, our patient due to financial constraints, was started on a TKI. After multidisciplinary discussion, our patient was initiated on pazopanib, and has remained free of disease at 22 months of follow-up.

Klatt et al. noted a better than expected survival in metastatic RCC when the primary tumor was <4 cm, with a median overall survival of 36 months (8). Similar findings were noted in a retrospective review of the Memorial Sloan Kettering and the International Metastatic RCC Database Consortium cytoreductive nephrectomy cohorts that showed improved overall survival in patients with metastatic clear-cell RCC, with primary tumor size ≤4 cm (2).

## Conclusions

SRM can occasionally exhibit aggressive features and present with metastatic disease. Sarcomatoid features may occur in metastasis without its presence in the primary tumor, likely due to denovo differentiation.

## Ethics Statement

The authors certify that informed consent was obtained from the patient, and all related information was deidentified prior to publication.
